# Susceptible and resistant olive cultivars show differential physiological response to *Xylella fastidiosa* infections

**DOI:** 10.3389/fpls.2022.968934

**Published:** 2022-09-20

**Authors:** Antony Surano, Raied Abou Kubaa, Franco Nigro, Giuseppe Altamura, Pasquale Losciale, Maria Saponari, Pasquale Saldarelli

**Affiliations:** ^1^Department of Soil, Plant and Food Sciences, University of Bari Aldo Moro, Bari, Italy; ^2^Institute for Sustainable Plant Protection, National Research Council (CNR), Bari, Italy; ^3^CRSFA-Centro Ricerca, Sperimentazione e Formazione in Agricoltura Basile Caramia, Locorotondo, Italy

**Keywords:** stomatal conductance, water potential, cultivar phenotyping, resistance, *Xylella*, olive

## Abstract

Olive quick decline syndrome (OQDS) is a severe disease, first described in Italy in late 2013, caused by strains of *Xylella fastidiosa* subsp. *pauca (Xfp)* in susceptible olive cultivars. Conversely, resistant olive cultivars do not develop OQDS but present scattered branch dieback, which generally does not evolve to severe canopy decline. In the present study, we assessed the physiological responses of *Xfp*-infected olive trees of susceptible and resistant cultivars. Periodic measurements of stomatal conductance (g_s_) and stem water potential (Ψstem) were performed using a set of healthy and *Xfp*-infected plants of the susceptible “Cellina di Nardò” and resistant “Leccino” and “FS17” cultivars. Strong differences in Δg_s_ and ΔΨstem among *Xfp*-infected trees of these cultivars were found, with higher values in Cellina di Nardò than in Leccino and FS17, while no differences were found among healthy plants of the different cultivars. Both resistant olive cultivars showed lower water stress upon *Xfp* infections, compared to the susceptible one, suggesting that measurements of g_s_ and Ψstem may represent discriminating parameters to be exploited in screening programs of olive genotypes for resistance to *X. fastidiosa*.

## Introduction

*Xylella fastidiosa* (*Xf*) is a gram-negative bacterium highly specialized to colonize xylem vessels of host plant species. *Xylella fastidiosa* is the causal agent of several important diseases such as Pierce's disease of grapevine (*Vitis vinifera*), citrus variegated chlorosis (CVC) in sweet orange, phony peach disease in peach (*Prunus persica*), coffee leaf scorch in *Coffea arabica*, almond leaf scorch in *Prunus dulcis*, plum leaf scald in *Prunus domestica*, and oleander leaf scorch in *Nerium oleander* (Janse and Obradovic, 2010). In the past decade, a strain of *Xylella fastidiosa* subsp. *pauca* (*Xfp*) has been accidentally introduced in Apulia, Southern Italy (Saponari et al., 2013), where it is the causal agent of a severe disease: the olive quick decline syndrome (OQDS). In olive groves, the bacterium was found to be efficiently transmitted by the predominant European xylem-sap feeder, the spittlebug *Philaenus spumarius*, responsible for the rapid spread of the OQDS (Martelli et al., 2016). Olive quick decline syndrome is characterized by scorching of leaves and scattered desiccation of branches and twigs. These symptoms usually start at the top of the olive canopy and expand to the rest of the crown, ultimately causing the death of the trees (Saponari et al., 2019). As general behavior of *Xf* in the host plants, bacterial cells attach to the vessel wall and, by multiplying, aggregate in a biofilm matrix composed of secreted nucleic acids, proteins, and exopolysaccharides (EPS) (Roper et al., 2007). These bacteria aggregates, together with the plant attempt to isolate the bacterium by tylose and gum production cause vessel occlusions and an impairment of water transport (Sun et al., 2013). This bacterial lifestyle suggests that in *Xf*-infected plants the soil-plant-atmosphere continuum (SPAC) is compromised. A scenario supported by several studies showed that a lower gas exchange (Gomes et al., 2003) and leaf water potential (Habermann et al., 2003a) occur in orange trees affected by CVC compared to healthy ones. Indeed, these latter authors suggested that xylem occlusion decreases the water supply to the leaves thus compromising the photosynthesis and the transpiration rates.

The stomatal conductance (g_s_) was used to discriminate resistant and susceptible cultivars of mango (*Mangifera* sp.) to the fungal vascular phytopathogen *Ceratocystis fimbriata* (Bispo et al., 2016). In this work measurements of g_s_ showed a positive correlation with the cultivars responses based on the disease indices and plant colonization.

Beside the vascular occlusion mechanism, a parallel role in inducing a water stress is played by the activity of *Xf* cell wall degrading enzymes. Their degradation activity on cell wall middle lamella of pit membranes contributes to reduce hydraulic conductivity in infected grapevines and to enhance the risk of embolisms (Fanton and Brodersen, 2021). Olive cultivars show different molecular and phenotypical responses to *Xfp*, with trees of the cultivars Leccino and FS17 showing resistance to the infection, i.e., with limited desiccation and low bacterial populations, while other cultivars such as *Ogliarola salentina* and Cellina di Nardò harbor higher bacterial load and develop severe symptoms, which in most cases led to the plant dieback (Boscia et al., 2017a). Although the mechanisms of resistance in Leccino and FS17 trees are still unclear, in a recent study, Leccino was found to be constitutively less susceptible to xylem cavitation than Cellina di Nardò, and was able to activate more efficient refilling mechanisms, thus rapidly restoring vessel's hydraulic conductivity (Sabella et al., 2019). Moreover, it has been hypothesized that the resistance of Leccino to *Xfp* could be related to its response to water stress, which could elicit defense pathways against *Xfp* mediated by sugars (De Pascali et al., 2022). In addition, molecular mechanisms entailing the plant cell wall receptors have been described in transcriptome studies (Giampetruzzi et al., 2016). The cell wall involvement is also supported by recent electron microscopy observations showing a higher pit membranes degradation in susceptible Cellina di Nardò compared to Leccino (Giampetruzzi et al., 2021; Montilon et al., 2022).

The present work aims to understand whether *Xfp* infections affect the physiological response of olives, in terms of impaired hydraulic conductivity and whether this response differs between one susceptible and two resistant cultivars. The study is propaedeutic to the application of these physiological measurements for the screening of a larger number of cultivars.

## Materials and methods

### Inoculation of olive cultivars with *Xfp*

In middle September 2020, 10 potted plants of the cultivars Cellina di Nardò, Leccino, and FS17, were used as recipient plants in a vector-mediated transmission experiment. Briefly, each plant was caged for 3 days with 10 specimens of *P. spumarius* previously confined for bacterial acquisition for 4 days on selected branches of an infected olive tree located in the demarcated area of Apulia. Real time PCR on 30 out of c. 300 insects after the acquisition period showed that the rate of insects positive to *Xfp* was 45% (data not shown). Transmission tests were performed in a screen-house located in the demarcated area (Vivai Arif Li Foggi, Lecce). Five plants were not caged and maintained under the same conditions, to serve as mock inoculated controls. At 6 and 12 months after the transmission experiments, each plant was individually tested for *Xfp*, by harvesting at least four leaves, whose petioles were excised and used for total DNA extraction using the commercial kit Maxwell (R) RSC PureFood GMO and Authentication Kit (Promega, USA). The presence of *Xfp* was checked by real-time qPCR using the assay developed by Harper et al. (2010).

### Plant materials and growing conditions

To assess if the colonization of the xylem tissues by bacterial cells and aggregates has a direct and measurable impact on the plant physiology, we selected systemically infected olive plants. More specifically, the plants that showed consistent qPCR positive results at 6 and 12 months, were considered systemically infected and retained for the stomatal conductance and stem water potential measurements. Four qPCR-positive plants and 4 mock inoculated plants were used for each cultivar. Measurements were started at 1 year post *Xfp*-transmission, when all plants were infected but visually symptomless. The selected olive plants were grown in pots (diameter 12 cm) containing a substrate composed by white peat (black peat (50:50) with a pH in H_2_O of 6.5 and an electrical conductivity of 0.4 dS/m). The substrate nutrient content was: 140, 100, and 180 mg/l of N, P_2_O_5_, and K_2_O, respectively. During the experiment, the plants were grown in a quarantine greenhouse at 25°C ± 2 and at relative humidity (RH) of 65% ± 10. The measurements of stomatal conductance and stem water potential were carried out at field capacity, corresponding to a soil water potential (Ψsoil) of −0.02 < Ψsoil < −0.03 MPa.

### Stomatal conductance measurements

The stomatal conductance (g_s_) was measured using a portable porometer (LI-600, LI-COR, Lincoln, Nebraska 68504, USA). For each plant, two mature leaves were selected and the mean value of the two g_s_ measurements calculated. Five independent measurements were carried out at mid-day starting in mid-September 2021, with 3–4 days intervals.

Differential stomatal conductance was calculates as followed:


$$\begin{array}{l} \Delta g_{s}=Hg_{s}-Ig_{s} \end{array}$$

where, *H*g_s_ and *I*g_s_ are the average of stomatal conductance of healthy and infected plants of the same cultivar, respectively.

### Stem water potential

Stem water potential (Ψstem) defined as the current water status of the plants was assessed twice, at the same time of the first and the fifth measurement of g_s_, using a Scholander-type pressure chamber Soil Moisture 3000 (Soil Moisture Corp., Goleta, CA, USA). Ψstem was measured on non-transpiring leaves, wrapped with aluminum foil for at least 1 h before the measurements, so that leaf water potential matched the stem water potential (Habermann et al., 2003a). Before the Ψstem measurement, the olive leaf was unlined from the aluminum foil, placed on the workbench and then deprived of a half of the leaf blade to facilitate the allocation inside the sample holder (Supplementary Figure 1). Values of Ψstem correspond to the mean of the measurements obtained from two leaves for each plant. Differential stem water potential (ΔΨstem) was calculated as follows:


$$\begin{array}{l} \Delta \text{Ψ}stem=H\text{Ψ}stem-\;I\text{Ψ}stem \end{array}$$

where, *H*Ψstem and *I*Ψstem are the average of the stem water potential of healthy (*H*) and infected (*I*) plants from the same cultivar, respectively.

### Experimental design and statistical analysis

Plants for the measurements of stomatal conductance and stem water potential were arranged in completely randomized design, with four replications for each cultivar. Data from all of the variables measured were assessed for normality and homogeneity of variance using the Bartlett test and subjected to the analysis of variance (ANOVA). When significant, the means of the treatments were compared using the Student-Newman-Keuls Test (*P* ≤ 0.05). The two groups (healthy and infected) were analyzed by the unpaired Student's *t*-test. Statistical analysis and Principal Component Analysis were performed with the RStudio software: Integrated Development for R, RStudio, PBC, Boston, MA.

## Results

### Real time qPCR

Six and twelve months after *Xfp* vector-mediated inoculation, positive qPCR reactions were obtained for the majority of the plants caged with infected specimens of *P. spumarius*. The Cq (quantification cycle) average values recorded for the infected plants selected in this study ranged from 20.24 for Cellina di Nardò, to 22.04 for Leccino and to 20.56 for FS17 (Supplementary Table 1), while mock-inoculated plants tested negative. Statistical analysis of the Cq-values obtained from infected plants did not show any difference.

### Stomatal conductance (g_s_)

**Figure 1:**
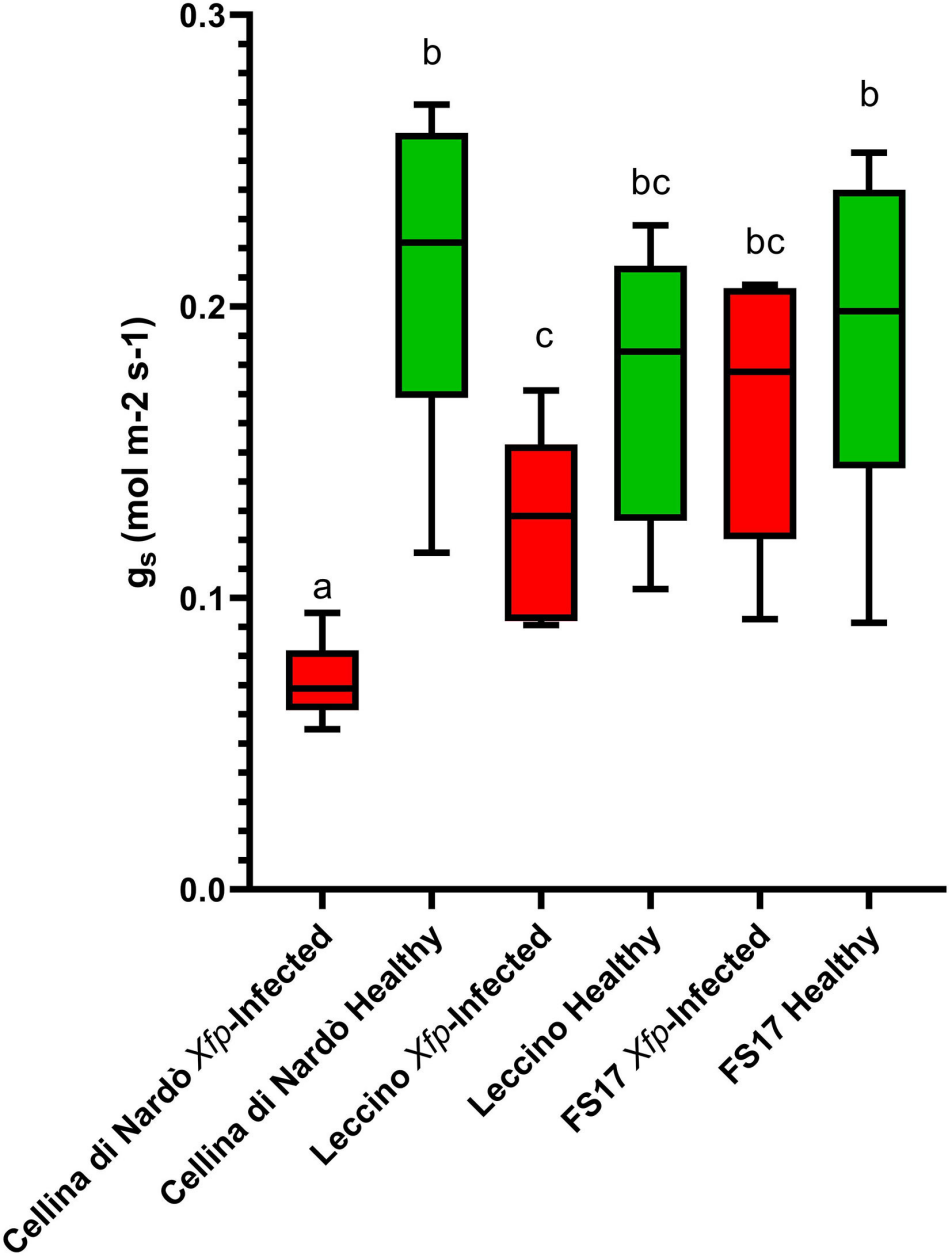
Box plot of stomatal conductance (g_s_) in healthy (green boxes) or *Xfp-*infected (red boxes) olives displaying the variation of data among the four infected or healthy plants of the three cultivars. Bars marked with the same letter are not statistically different, according to the Student–Newman–Keuls Test (*P* ≤ 0.05).

**Figure 2:**
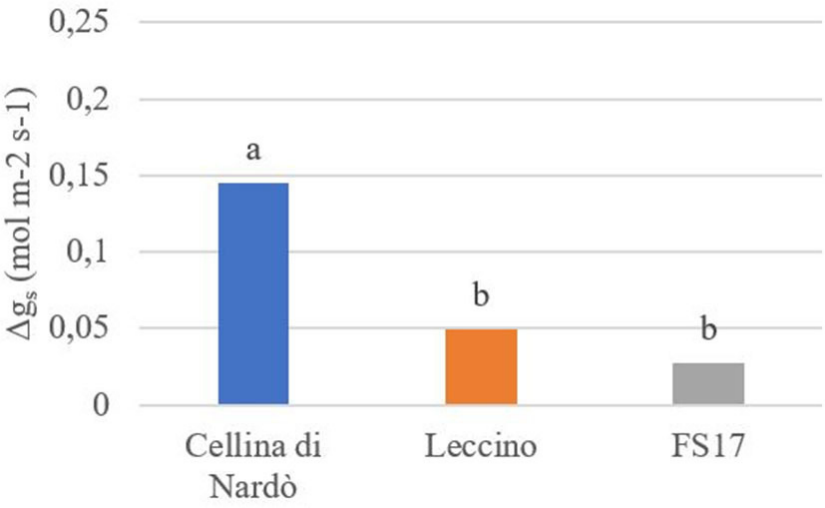
Differential stomatal conductance (Δg_g_) in healthy or *Xfp-*infected olives. Average values from four plants of the three cultivars are in the y-axis. Bars marked with the same letter are not significantly different, ac-cording to the Student–Newman–Keuls Test (*P* ≤ 0.05).

For each cultivar, a reduction of the stomatal conductance (mol m^−2^ s^−1^) was recorded in the *Xfp*-infected plants compared to the healthy ones. However, while g_s_ values for Cellina di Nardò were always significantly different between healthy and *Xfp*-infected plants, this was not the case for the plants of the two resistant cultivars. In Leccino, g_s_ of *Xfp*-infected plants were significantly different from healthy plants only at the first and second assessments. In FS17, *Xfp*-infected and healthy plants did not show differences in g_s_ during the whole experiment (Table 1). Collectively, the average g_s_ values of the healthy plants were not significantly different among the three tested cultivars (Figure 1, green boxes). Conversely, significantly different g_s_ average values were recorded between *Xfp*-infected plants of Cellina di Nardò in comparison with those of Leccino and FS17 (Figure 1, red boxes). Moreover, intra-cultivar comparison showed that stomatal conductances of infected Cellina di Nardò olives were significantly lower than that of the corresponding healthy plants, while these differences were not significant among infected and healthy plants of the other two cultivars (Figure 1). Concerning the differential stomatal conductance (Δg_s_) the data showed a constant trend during all assessments (Table 2). In particular, Cellina di Nardò showed a Δg_s_ significantly higher than Leccino and FS17 in all five assessments. The latter two cultivars did not differ from each other, with Leccino showing intermediate values, not statistically different from FS17 (Figure 2).

**Table 1:** Stomatal conductance (g_s_) of *Xfp*-infected or healthy plants of the three cultivars, during the five assessments.

**Assessment date**	**Infected or Healthy**	**g**_**s**_ **(mol m**^**−2**^ **s**^**−1**^**)**		
		**Cellina di Nardò**	**Leccino**	**FS17**
28 Sept.	Infected	0.094800	0.093581	0.177665
	Healthy	0.223491	0.149987	0.198447
		**	*	ns
1 Oct.	Infected	0.069214	0.134229	0.205255
	Healthy	0.24974	0.200009	0.227478
		***	**	ns
4 Oct.	Infected	0.067936	0.171211	0.207488
	Healthy	0.269279	0.227855	0.252709
		***	ns	ns
7 Oct.	Infected	0.068892	0.128206	0.147707
	Healthy	0.221905	0.184497	0.19759
		***	ns	ns
11 Oct.	Infected	0.054984	0.090632	0.092731
	Healthy	0.115406	0.103032	0.091379
		**	ns	ns

Values represents the average measurements of two leaves from four plants from each cultivar.

Significance level according to the Student's t-test (n = 4): ***P < 0.001; **0.001 < P < 0.01; *0.01 < P < 0.05; ns = not significant.

**Table 2:** Differential stomatal conductance (Δg_s_) among *Xfp*-infected or healthy plants of the three cultivars, during the five assessments.

**Cultivar**	Δ**g**_**s**_ **(mol m**^**−2**^ **s**^**−1**^)*				
	**28 Sept**.	**1 Oct**.	**4 Oct**.	**7 Oct**.	**11 Oct**.
Cellina di Nardò	0.128690a	0.180526a	0.201342a	0.153013a	0.060423a
Leccino	0.056406b	0.065780b	0.056643b	0.056291b	0.012399b
FS17	0.020782b	0.022223b	0.045221b	0.049883b	−0.001352b

Values represents the average measurements of two leaves from four plants for each cultivar.

*Means followed by the same letter in the column are not significantly different according to the Student-Newman-Keuls Test (P ≤ 0.05).

### Stem water potential (Ψstem)

**Figure 3:**
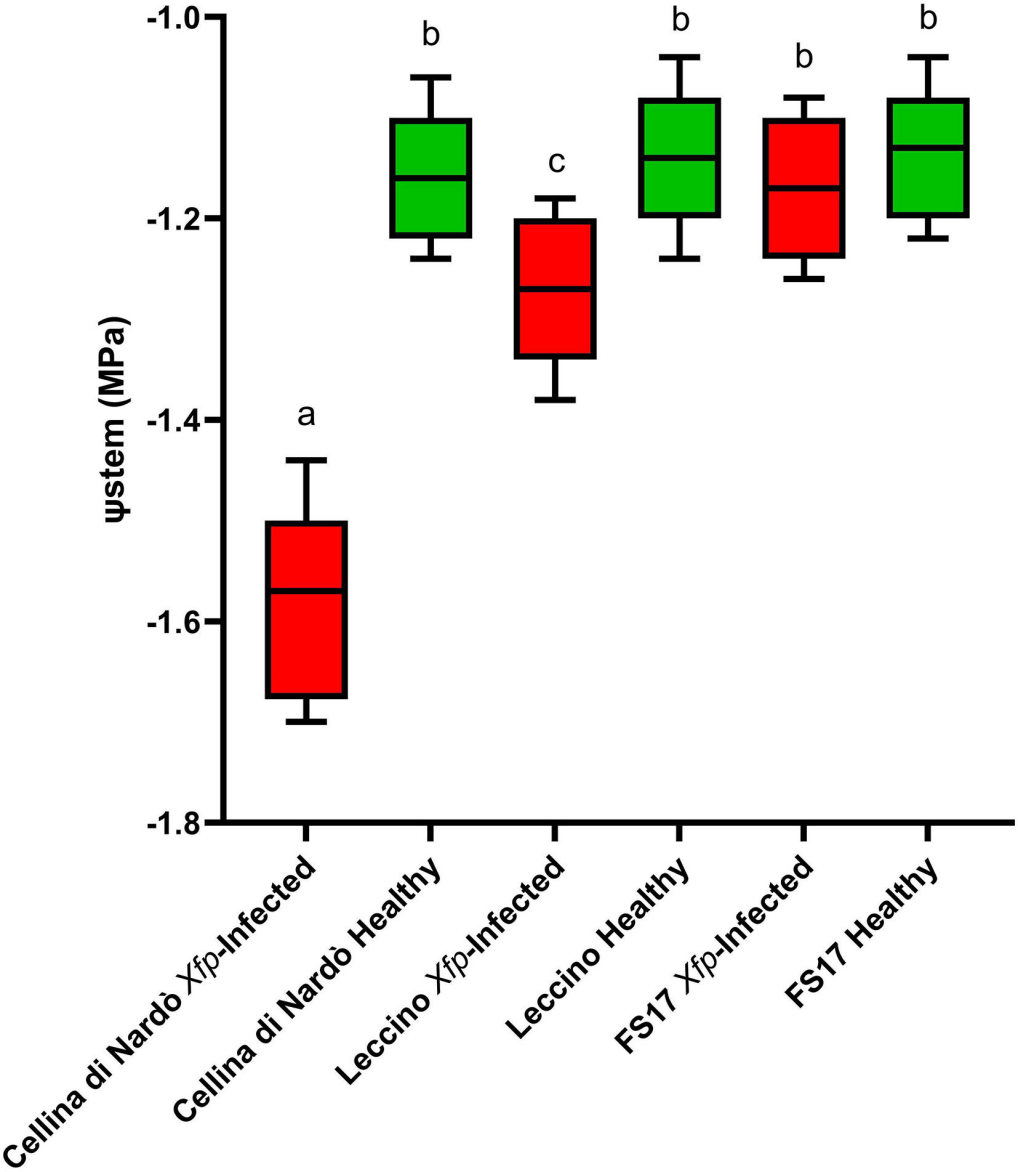
Box plot of stem water potential (Ψstem) in healthy (green boxes) or *Xfp*-infected (red boxes) olive plants displaying the variation of data among the four infected or healthy plants of the three cultivars. Bars marked with the same letter are not significantly different, according to the Student-Newman-Keuls Test (*P* ≤ 0.05).

**Figure 4:**
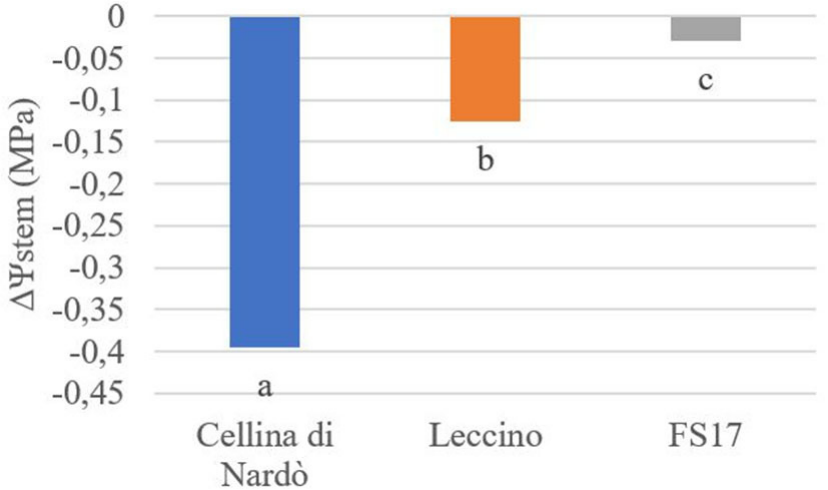
Differential stem water potential (ΔΨstem) in healthy or *Xfp*-infected olives. Average values from four plants of the three cultivars are in the y-axis. Bars marked with the same letter are not significantly different, according to the Student-Newman-Keuls Test (*P* ≤ 0.05).

Two measurements of the stem water potential (MPa) were taken throughout the trial. No difference of the average stem water potential was recorded among the healthy plants of the three cultivars (Figure 3, green boxes) while infected plants of the cultivar Cellina di Nardò had a significantly lower Ψstem (more negative) than Leccino and FS17 (Figure 3, red boxes). Infected plants of the cultivar Leccino showed a significant drop of the stem water potential in comparison with the healthy plants of the same cultivar, while this difference was not recorded among healthy and infected FS17 olives (Figure 3 and Table 3). A further evaluation showed that the three cultivars have clearly different values of differential stem water potential (ΔΨstem) (Figure 4). In particular, negative ΔΨstem values were observed in Cellina di Nardò, while limited differences of stem water potential were measured between healthy and *Xfp*-infected plants of the cultivar FS17 (i.e., ΔΨstem close to 0) and Leccino. Therefore, values of differential stem water potential were significantly different among the three cultivars. This trend was similar during both assessments (Table 4).

**Table 3:** Stem water potential (Ψstem) of *Xfp*-infected or healthy plants of the three cultivars, during the two assessments.

**Assessment date**	**Infected or Healthy**	Ψ**stem (MPa)**		
		**Cellina di Nardò**	**Leccino**	**FS17**
28 Sept.	Infected	−1.49	−1.20	−1.10
	Healthy	−1.09	−1.08	−1.08
		***	***	ns
11 Oct.	Infected	−1.67	−1.34	−1.24
	Healthy	−1.22	−1.20	−1.20
		***	**	ns

Values represents the average values of two leaves from four plants for each cultivar. During the two assessments, measurements were taken from four plants of the three cultivars.

Significance level according to the Student's t-test (n = 4): ***P < 0.001; **0.001 < P < 0.01; *0.01 < P < 0.05; ns = not significant.

**Table 4:** Differential stem water potential (ΔΨstem) among *Xfp*-infected or healthy plants of the three cultivars, during the two assessments.

**Cultivar**	***ΔΨ***stem (MPa)*	
	**28 Sept**.	**11 Oct**.
Cellina di Nardò	−0.40a	−0.45a
Leccino	−0.11b	−0.14b
FS17	−0.02c	−0.04c

Values represents the average values of two leaves from four plants for each cultivar.

*Means followed by the same letter in the column are not significantly different according to the Student-Newman-Keuls test (P ≤ 0.05).

**Figure 5:**
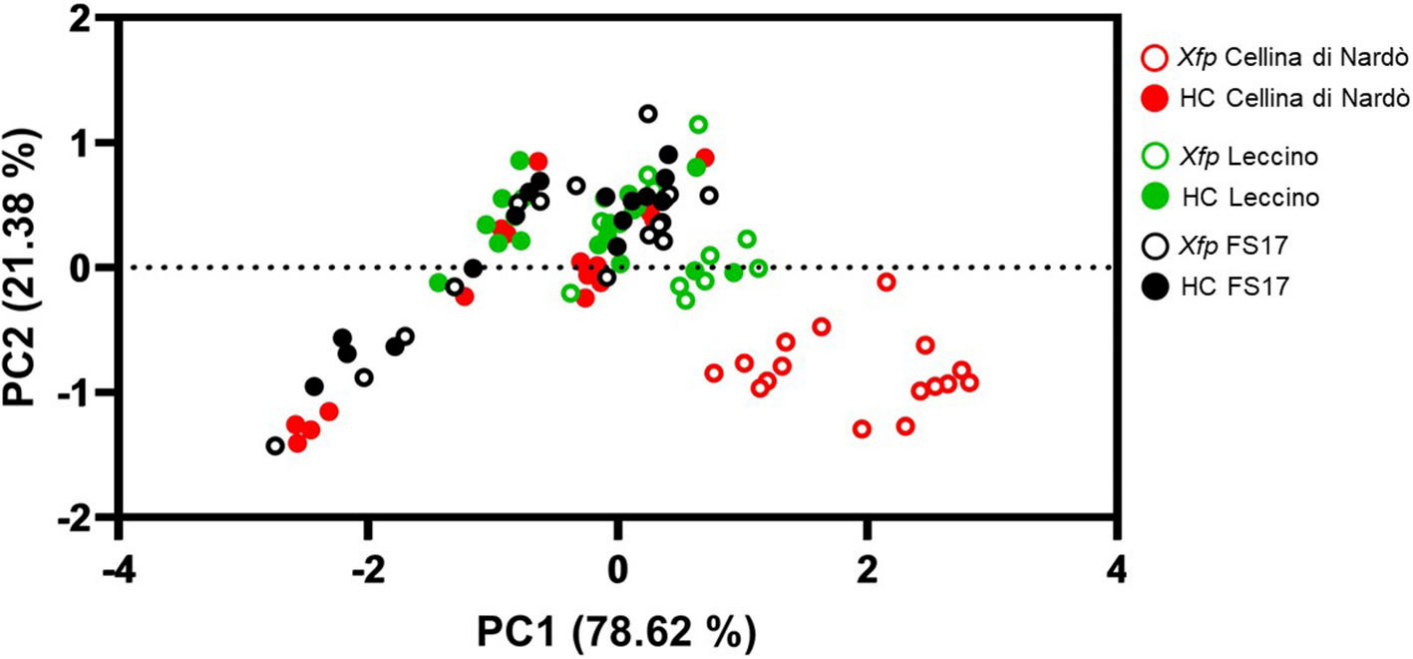
Principal Component Analysis using g_g_ (X-axis) and Ψstem (Y-axis) data from plants of the three cultivars measured throughout the whole experiment. Xfp and HC correspond to *Xfp*-infected and healthy plants, respectively.

A synoptical representation of the differential clustering of Cellina di Nardò in comparison with Leccino and FS17 is given by a Principal Component Analysis using g_s_ and Ψstem input data (Figure 5). A clear separation of infected olives of Cellina di Nardò from the plants of the other two cultivars was observed, demonstrating that *Xfp* infections determined measurable alterations of the physiological indexes in this cultivar, while these changes were less pronounced in the resistant cultivars FS17 and Leccino.

## Discussion

In the present work, measurements of g_s_ and Ψstem were carried out on healthy and *Xfp*-infected plants of susceptible and resistant olive cultivars, from which the differential stomatal conductance (Δg_s_) and the differential stem water potential (ΔΨstem) were calculated. Healthy plants showed similar g_s_ and Ψstem values, regardless of belonging to the resistant or susceptible cultivars. Conversely, lower stomatal conductance (g_s_) and more negative stem water potential (Ψstem) values were measured in *Xfp*-infected plants of the susceptible cultivar Cellina di Nardò, which were statistically different from those of the two resistant cultivars. Therefore, by comparing the differential stomatal conductance (Δg_s_) and stem water potential (ΔΨstem) values, we could categorize the plants belonging to the three cultivars. Under our experimental conditions, plants of the cultivar Cellina di Nardò suffered a severe hydraulic alteration when infected by *Xfp*, as showed by higher (Δg_s_) and negative ΔΨstem values. While both parameters were not much altered (i.e., close to 0) in infected plants of the two resistant cultivars. This result is in line with evidences from Habermann et al. (2003a,b) showing that a lower leaf water potential, indicative of an alteration in the water supply to the mesophyll, occurs in orange trees affected by CVC.

The outcomes of different studies in olives suggest an active host response in the cultivar Leccino to *Xfp* infections. Besides molecular mechanisms entailing the plant cell wall receptors and the immune response that have been described in transcriptome studies (Giampetruzzi et al., 2016), a better hydraulic management of the water stress induced by the pathogen has been also reported in this cultivar (Sabella et al., 2019). Indeed, these latter authors hypothesized that a more efficient refilling mechanism occurs in the plants of the cultivar Leccino, which ultimately allows it to mitigate the loss of hydraulic conductivity in infected plants, as a consequence of the vascular occlusions induced by the pathogen. In addition, changes in the stem hydraulic conductivity and consequently in the water transport occur in the infected plants as a direct impact of the bacterial dynamics and spread in the xylem tissues; more specifically as documented in grapevine, the degradation of Pit Membrane-middle lamellas by the bacterium cell wall degrading enzymes (Fanton and Brodersen, 2021) is one of the mechanisms underlining the loss of hydraulic conductivity. Similarly, a severe degradation of the pit membranes occurs in the susceptible cultivar Cellina di Nardò compared to the resistant cultivar Leccino (Giampetruzzi et al., 2021; Montilon et al., 2022). All together, these studies and the results herein reveal that *Xfp* infection determines physiological alterations in olives resembling a water stress, and that these changes are less pronounced in resistant cultivars. Furthermore, given that there is a positive correlation between stomatal conductance and crops yield (Pietragalla and Pask, 2012), and the positive correlation between stem water potential and olive yield (Fernandes-Silva et al., 2010; Moriana et al., 2012; Ghrab et al., 2013), the data herein collected support the speculation that resistant plants upon *Xfp* infections may remain productive. Considering that all infected plants, regardless of the cultivar, had similar Cq-values (i.e., similar bacterial load), the variations of the physiological parameters detected in our study are more likely the consequence of the differential host response to the bacterial infections rather than the direct influence of the pathogen abundance. The present data supports molecular evidences and extensive field surveys concluding that there is a different susceptibility of olive cultivars to *X. fastidiosa* infection (Habermann et al., 2003b; Boscia et al., 2017b; De Pascali et al., 2022). Indeed, this study is a preliminary step to introduce a phenomics approach in the screening of susceptible/resistant genotypes to *X. fastidiosa*. Such an approach aims to integrate diverse plant phenotypes to describe the interaction between genes and the environment (which is the pathogen's pressure, in our case) and to understand the impact of plant biological diversity in adaptation to biotic and abiotic stresses (Bilder et al., 2009; Furbank and Tester, 2011; Yol et al., 2015). To this end, our study gives a quantitative physiological tool to improve the phenotyping of the wide olive germplasm.

**Figure 6:**
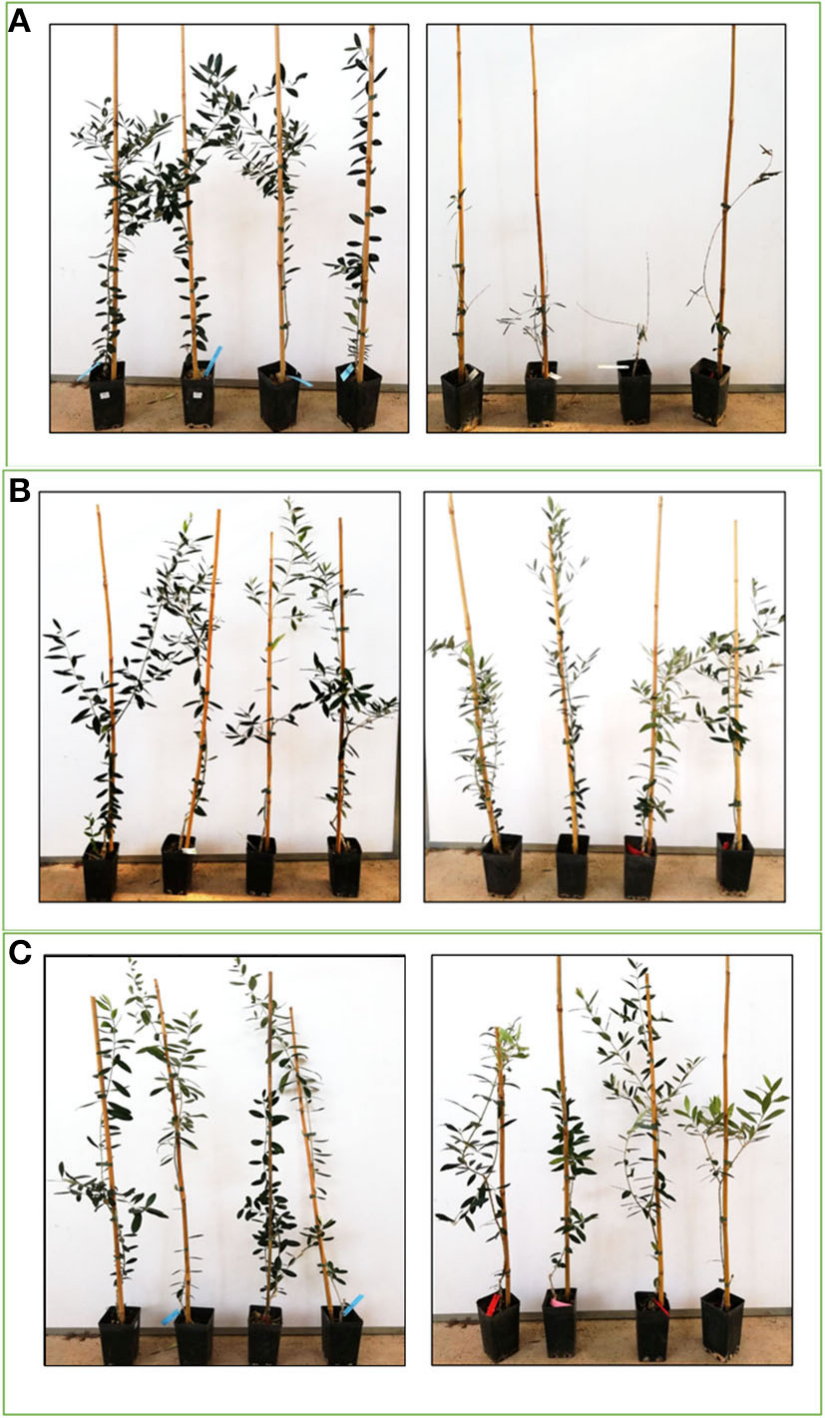
Olive plants used for the experiment showing symptoms 6 months after the end of the experiment (18 months after *Xfp* vector-transmission). Healthy plants on left and *Xfp*-infected plants on right. **(A)** Cellina di Nardò, **(B)** Leccino, and **(C)** FS17.

The long period of incubation of the *Xfp* infections in olive [EFSA Panel on Plant Health (PLH), 2015] implies that experiments for screening and differentiation of susceptible and resistant cultivars require long period of observations. In our study, we demonstrated that in asymptomatic infection, monitoring the physiological parameters can help to predict the behaviors of a given cultivar. Severe symptoms of desiccation were obtained on the *Xfp*-infected plants of Cellina di Nardò 18 months after vector transmission and 6 months after our measurements, while plants of FS17 and Leccino remained symptomless (Figure 6).

Our results confirm that *X. fastidiosa* infections in olive limit water supply to leaves, as indicated by the lower values of stomatal conductance and stem water potential detected in the infected plants compared to the healthy ones. To our knowledge this is the first study documenting physiological differences in olive trees infected by *X. fastidiosa*, and more importantly that physiological differences exist among these cultivars in response to the bacterium. Once this approach is validated in time-course experiments and on a larger panel of cultivars, it can be proposed as a complementary tool to support large-scale screening program of olive cultivars/genotypes for resistance to *X. fastidiosa*, contributing to reduce time, costs and infrastructures needed to perform such biological experiments. Several breeding programs aiming at developing new *X. fastidiosa* resistant genotypes have been started in the few past years, the assessment of the response to the bacterial infections is one of the major bottleneck in the selection process, increasing the time needed to fully assess the agronomical and productive performances of the newly developed genotypes. Perhaps, the possibility to rapidly assess the genotype response to bacterial infections, can contribute to speed up the breeding programs.

## Data availability statement

The raw data supporting the conclusions of this article will be made available by the authors, without undue reservation.

## Author contributions

AS and RAK performed the experiments. GA managed the plants. AS, PS, RAK, and MS planned trials and wrote the manuscript. PL and FN contributed to the discussion of the data and to the final editing. All authors listed have made a substantial, direct, and intellectual contribution to the work and approved it for publication.

## Funding

The work was funded by Regione Puglia, P.S.R. Puglia 2014/2020 - Misura 16 — Cooperazione - Sottomisura 16.2-Sostegno a progetti pilota e allo sviluppo di nuovi prodotti, pratiche, processi e tecnologie, Avviso Pubblico approvato con D.A.G. n. 194 del 12/09/2018, pubblicata nel B.U.R.P. n. 121 del 20/09/2018, and Project BIOSAVEX Olive Biodiversity for Saving Salento from Xylella.

## Conflict of interest

The authors declare that the research was conducted in the absence of any commercial or financial relationships that could be construed as a potential conflict of interest.

## Publisher's note

All claims expressed in this article are solely those of the authors and do not necessarily represent those of their affiliated organizations, or those of the publisher, the editors and the reviewers. Any product that may be evaluated in this article, or claim that may be made by its manufacturer, is not guaranteed or endorsed by the publisher.

## Supplementary material

Supplementary Figure 1 Sample preparation for Ψstem measurement.
