# Supplementary material for: Susceptible and resistant olive cultivars show differential physiological response to Xylella fastidiosa infections
Source: Front Plant Sci. 2022 Sep 20;13:968934. doi: 10.3389/fpls.2022.968934 (PMC9530328; doi:10.3389/fpls.2022.968934)
Supplement: Supplementary file 1 [file Table_1.DOCX]

Supplementary Table 1. Quantification cycles (Cq) of selected infected plants 12 month after Xfp vector-mediated inoculation

| Cultivar | Cq | | | |
| --- | --- | --- | --- | --- |
|  | Plant 1 | Plant 2 | Plant 3 | Plant 4 |
| Cellina di Nardò | 20,24 | 19,83 | 20,56 | 20,33 |
| Leccino | 21,61 | 23,22 | 22,16 | 21,17 |
| FS17 | 21,13 | 21,16 | 19,31 | 20,66 |
