# Supplementary figures and images for: Susceptible and resistant olive cultivars show differential physiological response to Xylella fastidiosa infections
Source: Front Plant Sci. 2022 Sep 20;13:968934. doi: 10.3389/fpls.2022.968934 (PMC9530328; doi:10.3389/fpls.2022.968934)

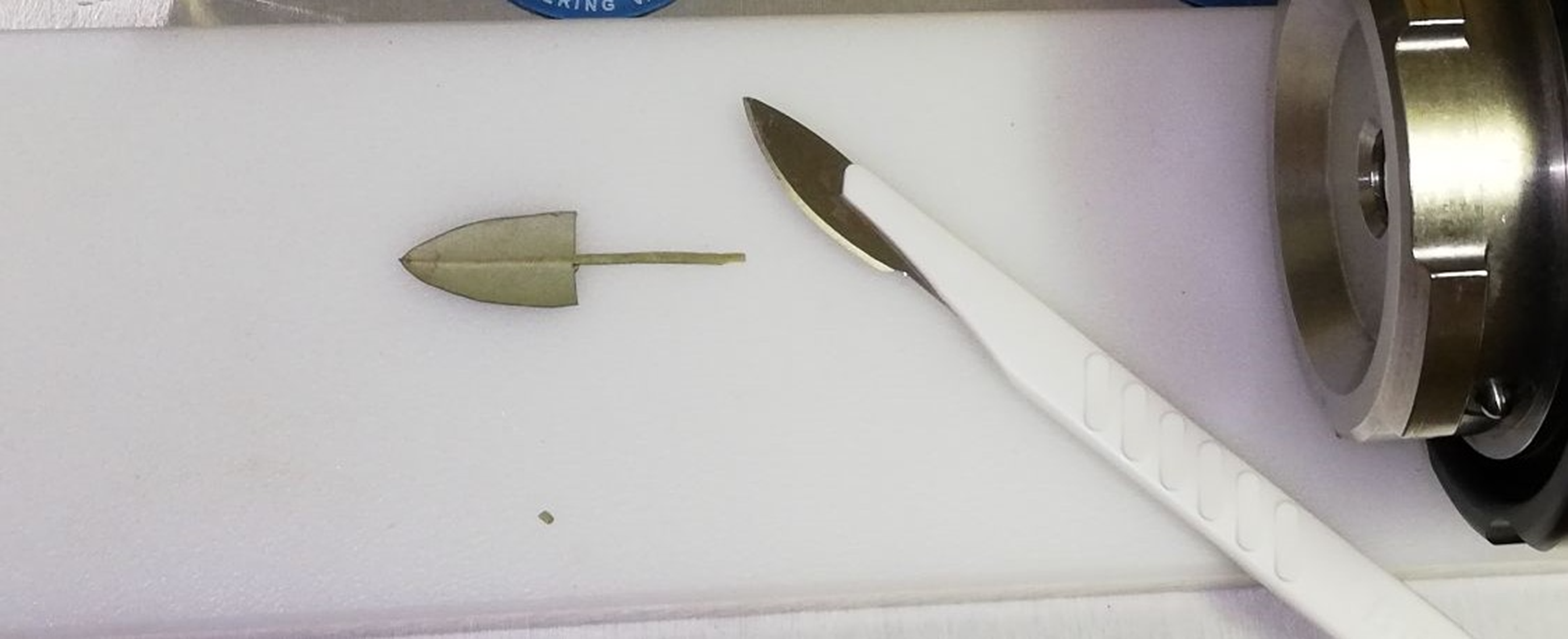

Supplement: Supplementary Figure 1 — Sample preparation for Ψstem measurement. [file Image_1.TIFF]
